# Supplementary material for: Identification of Benzophenone Analogs in Rice Cereal through Fast Pesticide Extraction and Ultrahigh-Performance Liquid Chromatography–Tandem Mass Spectrometry
Source: Foods. 2022 Feb 16;11(4):572. doi: 10.3390/foods11040572 (PMC8871057; doi:10.3390/foods11040572)
Supplement: Supplementary file 1 [file foods-11-00572-s001.zip › foods-1574143-supplementary.pdf]

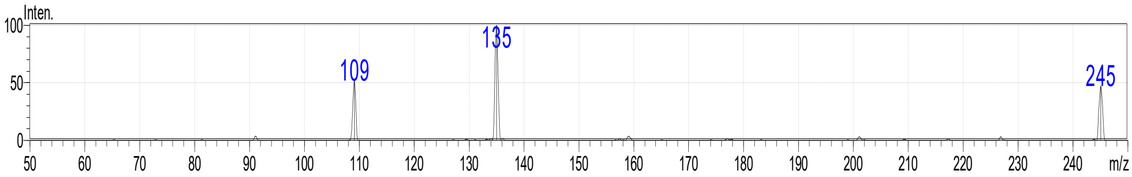

BP-2. Precursor ion: 245 (m/z); Collision energy:-15V

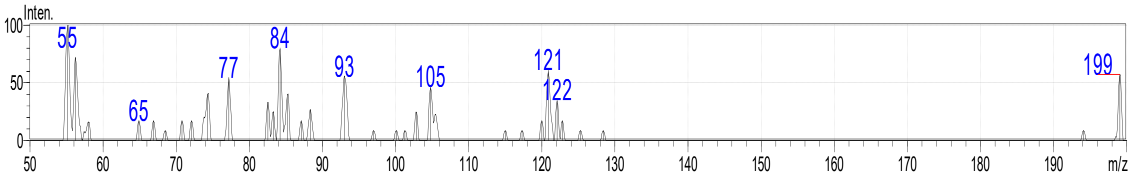

4-OHBP. Precursor ion: 199 (m/z); Collision energy: 25V

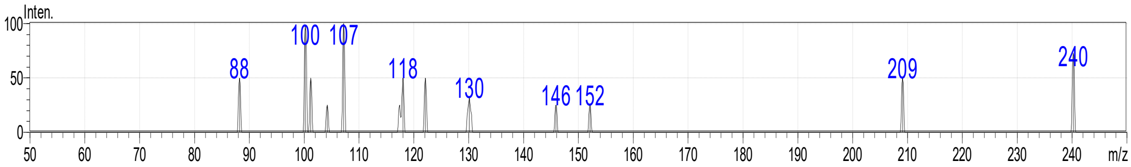

M2BB. Precursor ion: 240.25 (m/z); Collision energy: 20V

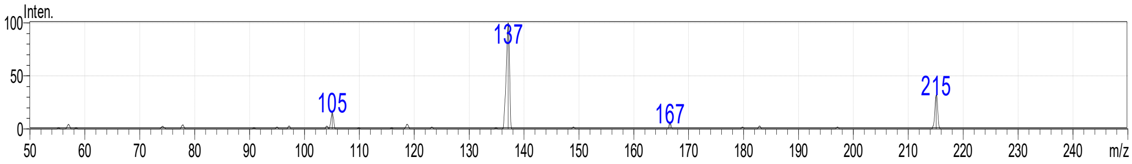

BP-1. Precursor ion: 215 (m/z); Collision energy: 20V

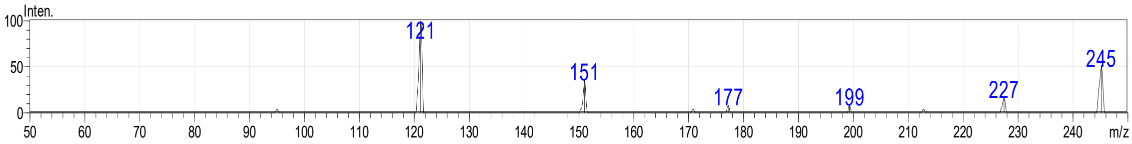

BP-8. Precursor ion: 245 (m/z); Collision energy: 15V

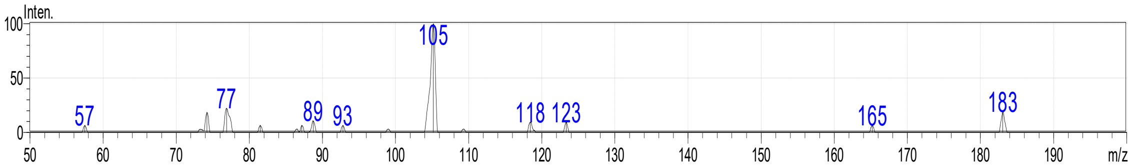

BP. Precursor ion: 183 (m/z); Collision energy: 20V

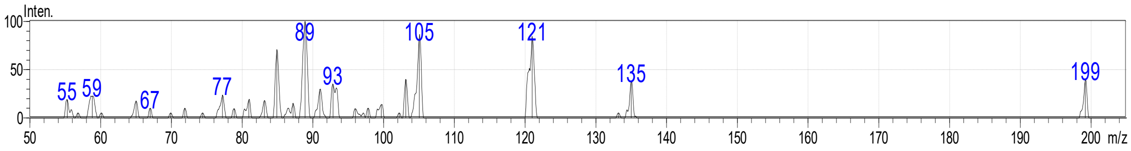

2-OHBP. Precursor ion: 199.2 (m/z); Collision energy: 25V

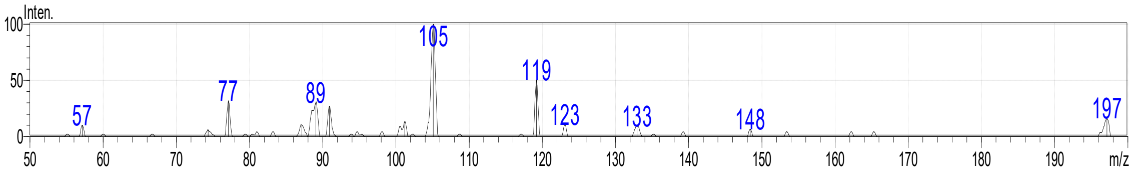

4-MBP. Precursor ion: 197 (m/z); Collision energy: 20V

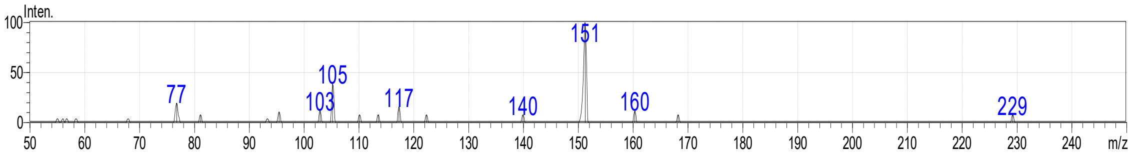

BP-3. Precursor ion: 229 (m/z); Collision energy: 25V

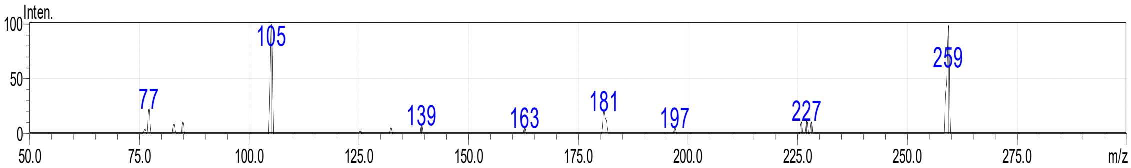

PBZ. Precursor ion: 259 (m/z); Collision energy: 15V

Figure S1. The respective mass spectra of the BP and nine BP analogs.
